# Indirect somatic embryogenesis of *Theobroma cacao* L*.* in liquid medium and improvement of embryo-to-plantlet conversion rate

**DOI:** 10.1007/s11627-018-9909-y

**Published:** 2018-06-04

**Authors:** Caroline Guillou, Audrey Fillodeau, Eric Brulard, David Breton, Simone De Faria Maraschin, Dorothée Verdier, Mathieu Simon, Jean-Paul Ducos

**Affiliations:** Nestlé Research & Development Centre Tours–Plant Science Research Unit, 101 avenue Gustave Eiffel BP49716, 37097 Tours CEDEX 2, France

**Keywords:** Cocoa, Propagation, High frequency somatic embryogenesis callus, Embryo-to-plantlet conversion rate, Myo-inositol

## Abstract

The establishment of cocoa embryogenic cell lines in liquid medium starting from high frequency somatic embryogenesis (HFSE) callus is described. The growth kinetics of the cultures during the multiplication and the expression steps conducted in 250 mL Erlenmeyer flasks were described for three genotypes selected for their agronomical traits (EET95, EET96, and EET103). The glucose and dissolved oxygen concentrations and the absorption of Murashige and Skoog medium macronutrients (nitrate, ammonium, potassium, sulfate, calcium, phosphorus, and magnesium) were monitored. The multiplication of the embryogenic calluses in a medium containing 2,4,5-trichlorophenoxyacetic acid (2,4,5-T) at 1 mg L^−1^, initiated with an inoculation density of 20 g L^−1^ of callus, was achieved. The growth rate was characterized by two phases, with the second being concomitant with a depletion of phosphorus and magnesium, and a decrease in the embryogenic potential of the callus. The expression of the callus embryogenic capacity was conducted in an auxin-free medium. The embryo production starting from 1 and 5 g L^−1^ inoculation densities was compared. When placed in the optimal expression conditions in flasks, 1 g of callus produced 1000 to 1500 embryos within 5 to 7 wk. Finally, two paths for improving the plantlet regenerative capacities of cocoa SE produced in liquid medium were identified. Supplementing the expression medium with myo-inositol used as an osmotic agent at a concentration of 50 g L^−1^ increased the embryo-to-plantlet conversion rate from 13–16% to 40–48%. A 6-wk culture of the embryos on a maturation medium in Petri dishes optimized their subsequent development into plantlets.

## Introduction

*Theobroma cacao* L. trees are grown in the humid tropics to produce cocoa beans. Four main countries produce 75% of the total cocoa beans: Ghana, Indonesia, Ivory Coast, and Nigeria (Food and Agriculture Organization, http://www.fao.org/faostat/en/#data/QC). In producing countries, cocoa is considered as a cash crop, but this is a crucial crop for the confectionary business, as it provides the key raw material for chocolate-based products. Nestlé utilizes about 12% of the world cocoa production.

Cocoa production from *T. cacao* trees is vulnerable in most producing countries due to the low income of farmers, diseases, pests, and adverse environmental conditions. To face these challenges, integrated propagation approaches based on somatic embryogenesis (SE), combined with traditional propagation techniques, have been described for the rapid dissemination of selected clones (Maximova *et al.* 2005; Miller 2009; Guillou *et al.* 2014). Due to SE, disease-free materials can be produced with an orthotropic growth habit, which the farmers are familiar with in some countries.

Somatic embryos can differentiate either directly from somatic cells, which undergo a minimum of proliferation (direct SE), or indirectly after extensive proliferation (indirect SE). However, the distinction between the two processes is not always clear. In coffee (*Coffea arabica* L.), Söndahl and Sharp (1977) preferred to use the terms low- and high frequency somatic embryogenesis (LFSE *versus* HFSE) to distinguish them. Low frequency somatic embryogenesis refers to the sporadic production of embryos from phenotypically indistinguishable embryogenic callus. In contrast, HFSE is characterized by the appearance of friable and highly embryogenic calluses containing pro-embryogenic masses (PEMs) (Söndahl *et al.* 1985). The specific nature of coffee HFSE callus allows the use of a liquid medium for embryogenic tissue proliferation (Zamarripa *et al.* 1991; Van Boxtel and Berthouly 1996; Etienne 2005). Commonly considered as indirect SE, HFSE cultures rapidly scale up the number of potential plants that can be produced, because it is possible to multiply the PEMs. Consequently, HFSE is generally preferred to LFSE (direct SE) for boosting mass propagation procedures (Etienne 2005). The multiplication of the embryogenic calluses (also referred as the “proliferation” or “maintenance” step) is generally conducted in an auxin-based medium. To initiate the regeneration of embryos (also referred as “expression” or “histo-differentiation” step), the calluses are transferred to an auxin-free medium, which allows them to express their embryogenic potential.

In the case of cocoa SE, most of the published literature is based on the progress made by Maximova *et al.* (2002), which applied to a particular case of SE that is classified as secondary embryogenesis (also referred to as “continuous,” “recurrent,” or “repetitive”) (Niemenak *et al.* 2008; Garcia *et al.* 2016). This method consists of culturing cotyledon fragments, from 1 to 2 cm long primary SE, for 2 wk on an auxin-based medium. After transfer to an auxin-free medium, each fragment produces 5 to 25 secondary SE within 1 y. Because the secondary SE emerges from a mass of non-embryogenic cells developing from the explants, this method corresponds *stricto sensu* to the HFSE. However, it leads to the regeneration of SE without the multiplication of embryogenic cells. To make the most of cocoa SE for creating higher mass propagation potential, a protocol was implemented based on obtaining and subculturing HFSE callus on a solid medium containing the auxin 2,4,5-trichlorophenoxyacetic acid (2,4,5-T) (Masseret *et al.* 2009).

As reported for many other plant species, converting the cocoa SE into whole plants is the most inefficient step of the process, and it depends on the genotype (Quainoo and Dwomon 2012; Maximova *et al.* 2014). A common approach for improving the SE quality is to create a partial dehydration. Numerous studies on conifers have shown the positive effect of increasing gelling agent concentrations and/or the duration of culture on a medium containing abscisic acid (ABA) (Klimaszewska and Smith 1997; Teyssier *et al.* 2011). To decrease their water content, olive (*Olea europaea* L.) SE are advantageously placed onto paper or cellulose acetate membranes (Cerezo *et al.* 2011). Restricting water uptake using high sucrose concentrations is efficient in alfalfa (*Medicago sativa* L.; Anandarajah and McKersie 1990) and avocado (*Persea americana* Mill.; Márquez-Martín *et al.* 2011), and supplementing the medium with PEG or sugar alcohols, such as mannitol or sorbitol in rice (*Oryza sativa* L.; Geng *et al.* 2008). Myo-inositol, as an osmotic agent, has been less often used to improve SE quality, and only in rose (*Rosa hybrida* L.; Castillón and Kamo 2002) and Norway spruce (*Picea abies* (L.) H. Karst.; Egertsdotter and Clapham 2015).

While several studies mention a low conversion of cocoa SE into plantlets, very few reports are available on the effects of individual components on SE quality in this species. Quainoo and Dwomon (2012) evaluated the effect of a range of ABA concentrations from 0 to 50 μM applied for a period of 0 to 6 wk. They did not observe any significant positive effects from these ABA treatments on the subsequent plantlet regeneration rates. Niemenak *et al.* (2015) reported that in medium containing sucrose at 60 g L^−1^ compared to 30 g L^−1^, maturated SEs contained higher levels of proteins involved in storage and tolerance to desiccation.

An HFSE procedure for establishing cocoa embryogenic cell lines in liquid medium is described in this study. The growth kinetics and the nutrient uptake during the multiplication and expression steps in liquid medium were characterized for two clones selected for their agronomic value. The study also focused on how to improve the conversion of cocoa SE into viable plantlets by assessing the effects of increasing the osmolality of the medium during the expression step in flasks, and the effects of the duration of the culture on a maturation medium in Petri dishes. After these different treatments, the embryo-to-plantlet conversion rates on germination medium were compared.

## Materials and Methods

### Plant material

In this study, four genotypes were chosen: the clone Scavina 6 (SCA6) from the genetic cluster Contamana, and the clones EET95, EET96, and EET103 from the genetic cluster Nacional (Motamayor *et al.* 2008). Contrary to the SCA6, which is a clone often used for studies due to its high embryogenic capacity (Li *et al.* 1998; Maximova *et al.* 2002), EET95, EET96, and EET103 were chosen for their Ariba flavor and agronomical traits.

*In vitro* tissues were developed from immature floral buds collected in August 2013, from trees grown in fields in the Nestlé experimental farm in Chollo, Ecuador.

### Induction of primary SE and HFSE callus

Primary SE were collected from staminodes following the protocol of Li *et al.* (1998). The explants were placed on a PCG medium gelled with Gelrite® at 3 g L^−1^ for 2 wk. The PCG medium contained 2 mg L^−1^ 2,4-dichlorophenoxyacetic acid (2,4-D), 5 μg L^−1^ thidiazuron (TDZ), and 50 mg L^−1^ isopentenyl adenine (IP). The complete composition of the medium is listed in Table 1. The explants were placed in a dark room at 25°C in 55 mm Petri dishes. After 2 wk, the explants were transferred to SCG medium, which incorporated 2 mg L^−1^ 2,4-D and 0.25 mg L^−1^ kinetin. The explants were then placed on the auxin-free CC2 medium for 12 wk.

**Table 1.** Media composition [^1^Driver and Kuniyuki (1984); ^2^McCown and Lloyd (1981); ^3^Murashige and Skoog (1962)]

	Induction		Multiplication	Expression	Maturation	Germination
Code	PCG	SCG	CC21	CC2	G80	ENR8
Macronutrients	DKW^(1)^	WPM^(2)^	MS^(3)^	MS^(3)^	MS^(3)^/2	MS^(3)^
Micronutrients	DKW^(1)^	WPM^(2)^	DKW^(1)^	DKW^(1)^	MS^(3)^	DKW^(1)^
Vitamins (mg L^−1^)						
Myo-inositol	200.0	100.0	100.0	100.0	100.0	100.0
Nicotinic acid	1.0	1.0	1.0	1.0	0.5	1.0
Thiamine	2.0	10.0	2.0	2.0	0.7	2.0
Pyridoxine-HCl		1.0			0.5	
Amino acids (mg L^−1^)						
Glycine	2.0	2.0	2.0	2.0	2.0	2.0
L-Lysine			0.4	0.4	0.4	0.4
L-Leucine			0.4	0.4	0.4	0.4
L-Arginine			0.4	0.4	0.4	0.4
L-Tryptophane			0.2	0.2	0.2	0.2
Glutamine	250.0					
Plant growth regulators (mg L^−1^)						
2,4,5-T			1.000			
2,4-D	2.000	2.000				
Naphthaleneacetic acid					0.010	
Kinetin		0.250				
Thidiazuron	0.005					
Isopentenyl adenine	50.000				0.200	
Adenine-H_2_SO_4_			0.250	0.025		
Abscisic acid					1.000	
Gibberellic acid					0.020	
Coconut milk (mL L^−1^)		50.0				
Other (g L^−1^)						
Activated charcoal					1	
Glucose	20	20	30	30	40	30
Gelrite®	3	3			3	3
pH	5.6	5.6	5.6	5.6	5.8	5.8

**Figure 1.**
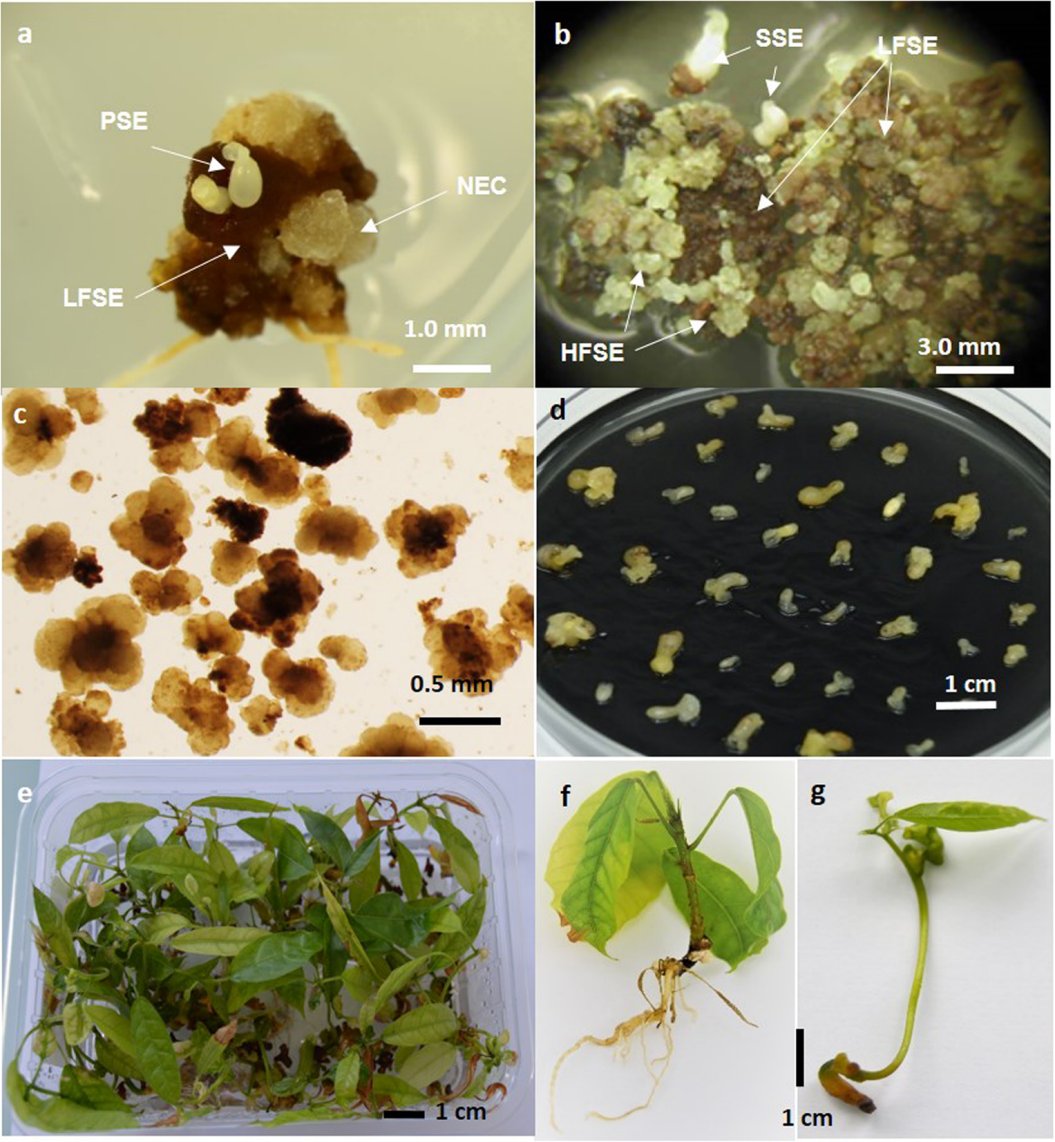
The different steps for *Theobroma cacao* L*.* propagation by high frequency somatic embryogenesis (HFSE). (*a*) Induction of primary SE (PSE, primary somatic embryos; LFSE, low frequency somatic embryogenesis calluses; NEC, non-embryogenic calluses); (*b*) induction of HFSE callus (SSE, secondary somatic embryos; LFSE, low frequency somatic embryogenic calluses); (*c*) multiplication: embryogenic clumps in liquid medium; (*d*) expression: an embryo population at the end of the step; (*e*) germination: plantlets in germination boxes; (*f*) normal plantlets; (*g*) abnormal plantlets.

Three to 4 mo after disinfection, primary somatic embryos (PSE) started to develop from primary callus (Fig. 1*a*). Embryos at the torpedo-stage were finely cut with a scalpel and the fragments placed on a CC21 medium containing the auxin 1 mg L^−1^ 2,4,5-T, and 0.25 mg L^−1^ adenine. Every 6 wk, the embryo fragment calluses were subcultured onto fresh medium for a maximum period of 8 mo. During this time, HFSE callus started to appear.

### Multiplication of the HFSE callus in liquid medium

The establishment of embryogenic cell lines in liquid medium was initiated by selecting HFSE calluses (Fig. 1*b*). From 0.05 to 0.10 g fresh weight (FW) of the HFSE callus was transferred into 10 mL of the multiplication medium (CC21) contained in 25-mL Erlenmeyer flasks (cycle 1). The medium was renewed every 3 wk by doubling the volume of the suspensions and transferring them into larger flasks as follows: 20 mL in 50-mL flasks at 3 wk (cycle 2), 50 mL in 100-mL flasks at 6 wk (cycle 3), and 100 mL in 250-mL flasks at 9 wk (cycle 4). After 12 wk of culture in liquid medium, the biomass was collected by filtration through a 50-μm filter. The calluses were selected and transferred into 100 mL of the CC21 medium with an inoculation density of 20 g L^−1^ (cycle 5). Thereafter, the embryogenic cell lines (Fig. 1*c*) were subcultured every 3 wk into 250-mL flasks by transferring 2 g of callus into 100-mL of fresh CC21 medium. The suspensions were cultured on an orbital shaker (New Brunswick™ Innova® 2300, Nijmegen, The Netherland) at 120 rpm placed in a dark room at 25°C.

Growth kinetics of cell lines after eight multiplication cycles were established by measuring various parameters after 0, 1, 3, 5, and 7 wk of culture (named respectively in the results and discussion T0W, T1W, T3W, T5W, and T7W). The parameters measured were biomass concentration (FW), glucose concentration, dissolved oxygen concentration (DO_2_), pH, osmolality, conductivity, and MS macronutrient concentrations (nitrate, potassium, ammonium, sulfate, calcium, phosphorus, and magnesium). The embryogenic potential of the calluses, collected at different time periods of a multiplication cycle, was measured by counting the number of embryos produced per gram of callus 4 wk after their transfer into the CC2 expression medium.

### Expression of the callus embryogenic potential in liquid medium

To start the regeneration of the embryos, embryogenic calluses were transferred into 250-mL flasks containing 100 mL of expression medium (CC2). The suspensions were cultured in a dark room at 25°C at 120 rpm.

Growth kinetics was established using calluses developing from the same embryogenic cell lines as the ones studied for the multiplication kinetics. The expression starting from two different inoculation densities, 1 and 5 g FW L^−1^, was compared. The cultures were analyzed after 1, 3, 5, and 7 wk (named in the results and discussion T1W, T3W, T5W, and T7W) for biomass, glucose, DO_2_, and MS ion concentrations.

To study the effects of medium components on tissue osmolality, two experiments were done. In the first experiment, the effects on clone EET103 in the expression step of 15, 30, and 45 g L^−1^ myo-inositol (corresponding to a tissue osmolality level of 337, 441, and 542 mOsm kg H_2_O^−1^, respectively) were assessed in CC2 medium containing glucose at 30 g L^−1^ (corresponding to a tissue osmolality level 261 mOsm kg H_2_O^−1^). The second experiment compared the expression of clones SCA6 and EET103 in medium supplemented with myo-inositol at 50 g L^−1^, after 4 wk of culture, and in a medium containing sucrose at 30 or 80 g L^−1^(tissue osmolalities differed from 193 to 556 mOsm kg H_2_O^−1^).

### Embryo-to-plantlet conversion on solid medium

After collection from the flasks, the embryos at the torpedo-stage were individually transferred into 90 × 20-mm Petri dishes containing G80 maturation medium (Fig. 1*d*) containing 1 mg L^−1^ ABA and 1 g L^−1^ charcoal (Lopez-Baez *et al.* 1993). Each Petri dish was inoculated with 30–40 embryos. The embryos were incubated in a dark room at 25°C.

After 0, 3, 6, and 9 wk of maturation (named in the results and discussion T0W, T3W, T6W, and T9W), the embryos were individually subcultured onto ENR8 germination medium in 145 × 100 × 60-mm styrofoam boxes (Dominique Dutscher S.a, ref. 017002, Issy les moulineaux, France). The embryos were germinated with a dark/light cycle of 12/12 h at a light intensity of 65 μmol m^−2^ s^−1^ with two LED Tubes Green Power Deep Red/White/Medium blue, 18 W, 24 μmol S^−1^, (Philips Lighting, Zurich, Switzerland) in a culture room at 25°C.

The embryo-to-normal plantlet conversion rates (%) were measured after 8 wk by counting the embryos that had developed into normal plantlets (Fig. 1*e*). The normal plantlets had a rooting apex, and, at least, a 2-cm stem height, and one pair of true leaves with a minimum length of 1 cm (Fig. 1*f*). These fully developed plantlets with standard characteristics were able to be successfully acclimated to the greenhouse. In some experiments, the number of embryos was counted, which had developed abnormal plantlets characterized by thin shoots, and dark, thickened, and stunted leaves (Fig. 1*g*). These types of plantlets are not able to survive to the transfer into the greenhouse.

All the chemicals are from Duchefa, Haarlem, The Netherlands and Petri dishes from VWR, Fontenay-sous-Bois, France. The media pH was adjusted with either 0.1 or 1 N hydrochloric acid and potassium hydroxide. Media were autoclaved at 121°C for 20 min and glass vessel at 121°C for 30 min in a Lequeux autoclave, Paris, France.

### Analytical measurements

*Glucose*: Glucose level in the media was measured using a high-performance liquid chromatograph (Dionex, IC5000, Thermo Fisher Scientific®, Waltham, MA). The separation was achieved using a 4 × 150-mm ion exchange column (Dionex CarboPac, PA10, Thermo Fisher Scientific®).

*Dissolved oxygen concentration*: Oxygen was non-invasively measured by using PSt3 sensor spots placed on the bottom of the flasks along with an optic meter (Firebox 3, PreSens®, Regensburg, Germany).

*Conductivity and pH*: Both the conductivity and pH values were measured directly in the cell culture broth using a digital apparatus (Model 3540, Jenway®, Chelmsford, UK).

*Osmotic potential*: The osmolality was measured in milliosmoles per kilogram of H_2_O using a micro-osmometer (Model 6/6M, Löser®, Berlin, Germany). The embryo osmolality was determined as following the method of Teyssier *et al.* (2011): Around 10 embryos were stored at − 20°C and thawed at 50°C for 10 min. After centrifugation at 8000×*g* for 15 min, the osmolality was measured in 100 μL of the supernatants.

*Macronutrients*: Residual concentrations of MS ions in the spent medium were measured by conventional methods (Laboratoire de Touraine, Tours, France). Nitrate and ammonium levels were determined by flow analysis and spectrometric detection according respectively NF-EN ISO 13395:1996 and NF-EN ISO 11732 (13395:2005). Sulfate level was determined by ionic liquid chromatography according NF-EN ISO 10304-1:2007/2010, and the levels of cations potassium, calcium, phosphorus, and magnesium were determined by inductively coupled plasma optical emission spectrometry according NF-EN ISO 11885:2007. All the protocols are available on the website https://www.boutique.afnor.org/.

*Water content (WC)*: After collection from the flasks, embryos were weighed to determine their FW before being dried in an oven (UN55, Memmert®, Schwabach, Germany) at 90°C for 24 h. Just after being taken out of the oven, the samples were weighed again to determine their dry weight (DW). The WC was calculated using the following equation: WC (%) = (DW / FW) × 100.

### Data analysis

Statistical analyses, including ANOVA using the general linear model (GLM), and tests to compare treatment averages, were performed using Minitab® 17.3.1 (Minitab® Inc., State College, PA). The relevance and ranking factors were calculated by comparing the averages using the Tukey test at the 5% threshold.

## Results and Discussion

### Induction of HFSE callus

Young secondary SE (SSE) became observable after 1 mo of culture on the CC21 solid medium containing 1 mg L^−1^ 2,4,5-T (Fig. 1*b*). Depending on the genotype, small embryogenic calluses (HFSE) appeared within 6 to 10 mo (data not shown). They were characterized by a granular and friable appearance and a yellowish color (Fig. 1*b*). Secondary SE and HFSE calluses did not develop directly from the fragments of primary SE, but from brown to dark brown callus.

The differences observed between the occurrence of cocoa secondary SE and embryogenic callus are very similar to the differences reported in Arabica coffee, between the LFSE and HFSE processes (Söndahl *et al.* 1985; Etienne 2005). In this species, SE takes place in two waves in leaf explant cultures. The first wave (LFSE) corresponds to the sporadic and transient production of isolated SE within 2 to 3 mo, and the second wave (HFSE) corresponds to the production of very embryogenic callus within 5 to 10 mo.

Importantly, the occurrence of cocoa HFSE callus was found only if fragments were isolated from the 2–3-mm torpedo-stage, rather than from 1-cm cotyledonary-stage embryos, which other authors used as starting material to obtain secondary SE (Maximova *et al.* 2002; Garcia *et al.* 2016).

In the frame of a core collection establishment, it was observed that almost all of the genotypes that are reactive to primary SE also respond positively to secondary SE, which confirms the results of Maximova *et al.* (2005), Masseret *et al.* (2009), and Guillou *et al.* (2014). However, there were some genotypes that produced primary and secondary SE very well, but for which obtaining and selecting HFSE calluses remained a long and laborious process. It is estimated that 50 to 60% of the genotypes gave rise to HFSE callus using this protocol (data not shown).

### Multiplication of the HFSE callus in liquid medium

Establishment of embryogenic cell lines. The HFSE calluses must be transferred into a liquid medium with a sufficiently high inoculation density of 5 to 10 g FW L^−1^ as a minimum. Indeed, with lower inoculum, the suspensions frequently tended to differentiate totally into embryos despite the presence of the 2,4,5-T in the CC21 medium. For this reason, and because of the limited quantity of available HFSE calluses, the cell lines have to be initiated in small volumes of medium, generally by transferring 0.05 to 0.10 g FW of selected callus into 10 mL of medium. Next, the volume of the medium was doubled every 3 wk until 12 wk. At this time (cycle 5), the biomass consisted of a mixture of secondary SE and calluses of various types. To initiate the maintenance of the embryogenic cell lines, the friable and yellowish calluses were selected once again if necessary and transferred into 250-mL flasks with an inoculation density of 20 g FW L^−1^ to limit the differentiation of embryos and to maintain HFSE calluses. The cell lines were composed of large aggregates, ranging from 0.3 to 1.5 mm (Fig. 1*c*).

**Figure 2.**
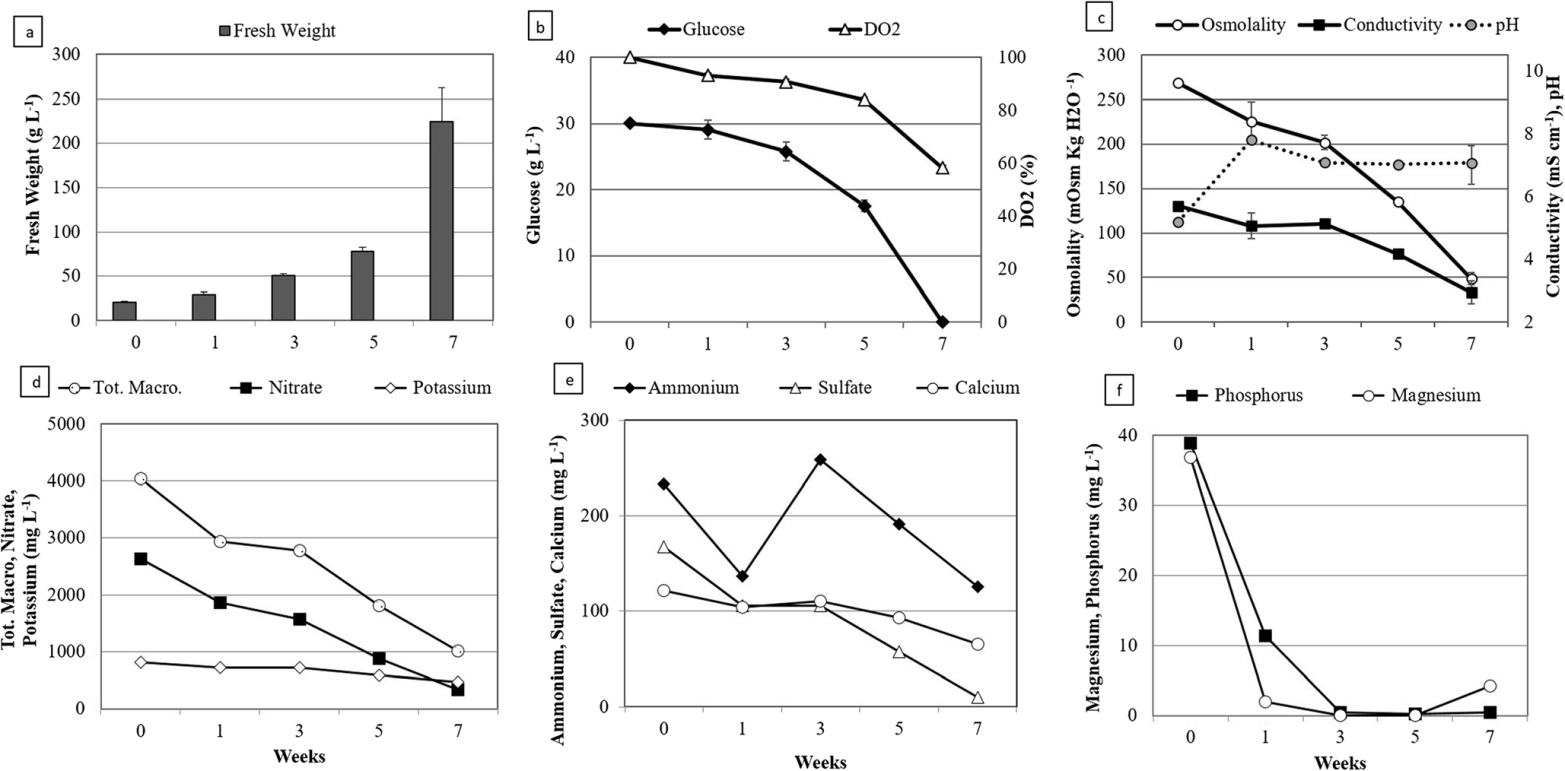
Multiplication step: growth kinetics and nutrient uptake of *Theobroma cacao* L*.* cv. EET96 embryogenic cell line. (*a*) Fresh weight (g L^−1^); (*b*) glucose concentration (g L^−1^) and DO_2_ (%); (*c*) tissue osmolality, conductivity, and pH; (*d*) total macronutrients, nitrate, and potassium concentrations (mg L^−1^); (*e*) ammonium, sulfate, and calcium concentrations (mg L^−1^); (*f*) phosphorus and magnesium concentrations (mg L^−1^). For *a*, *b*, and *c*, the data represent the averages of four replicates, each one being a 250-mL flask containing 100 mL of CC21 medium initiated with an inoculation density of 20 g L^−1^. *Bars* represent standard errors unless too small to visualize. For *d*, *e*, and *f*, measurements were taken after combining the spent medium of four flasks.

**Figure 3.**
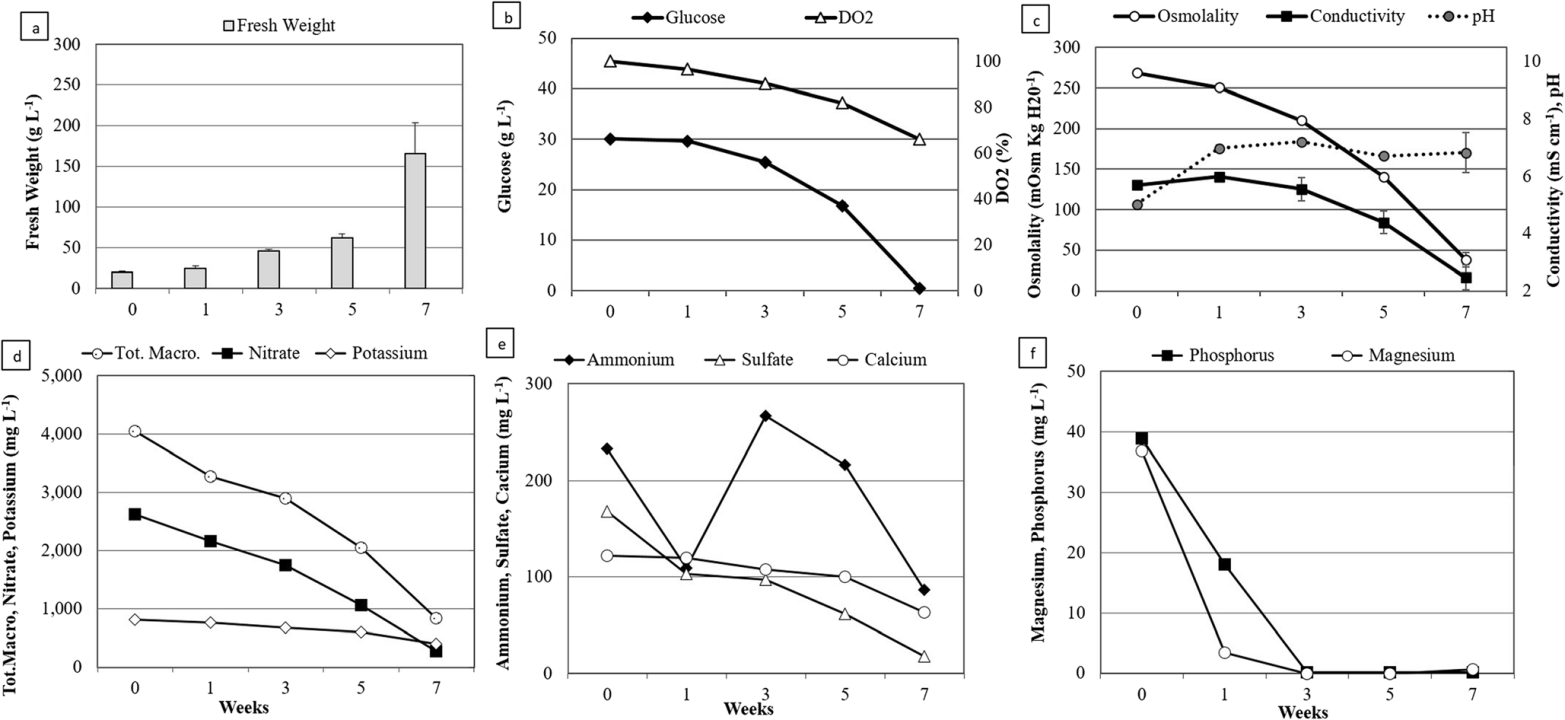
Multiplication step: growth kinetics and nutrient uptake of *Theobroma cacao* L*.* cv. EET103 embryogenic cell line. (*a*) Fresh weight (g L^−1^); (*b*) glucose concentration (g L^−1^) and DO_2_ (%); (*c*) tissue osmolality, conductivity, and pH; (*d*) total macronutrients, nitrate, and potassium concentrations (mg L^−1^); (*e*) ammonium, sulfate, and calcium concentrations (mg L^−1^); (*f*) phosphorus and magnesium concentrations (mg L^−1^). For *a*, *b*, and *c*, the data represent the averages of four replicates, each one being a 250-mL flask containing 100 mL of CC21 medium initiated with an inoculation density of 20 g L^−1^. *Bars* represent standard errors unless too small to visualize. For *d*, *e*, and *f*, measurements were taken after combining the spent medium of four flasks.

Growth kinetics. Typical profiles of the growth and of substrate consumption of EET96 and EET103 embryogenic cell lines are shown in Figs. 2 and 3. The biomass concentrations ranged from 20 up to 60–80 g FW L^−1^ within 5 wk. During this period, the growth was strictly linear and followed the equations y = 11.14x + 20 with an R^2^ value of 0.99 for EET96, and y = 8.39x + 20 with an R^2^ value of 0.98 for EET103. Afterwards, the growth rates increased strongly between T5W and T7W, as the biomass concentration was threefold higher within only 2 wk. It reached a final value of 224 and 166 g FW L^−1^ at T7W for the clones EET96 and EET103, respectively (Figs. 2*a*, 3*a*). By T7W, the glucose was completely consumed from the medium (Figs. 2*b*, 3*b*). Because oxygen can be a limiting factor in liquid medium, the cultures were checked for hypoxia. The DO_2_ did not drop below 60% throughout the cultures. In another experiment (data not shown), after shaking the cultures was stopped, the DO_2_ critical value under which the consumption rate in O_2_ decreases was determined and indicated hypoxia regime. For the cocoa calluses cultured in suspensions, this value lies between 35 and 45%. Consequently, it can be assumed that the DO_2_ was not a limiting factor during the culture period shown in the Figs. 2 and 3. The pH started at 4.6 in T0W before reaching values ranging from 6.0 to 7.0 (Figs. 2*c*, 3*c*). The tissue osmolality decreased from an initial value of 269, to values close to 50 mOsm kgH_2_O^−1^ at the end of the culture period, with the medium conductivity dropping from 5.2 to 1.9–2.4 mS cm^−1^.

The sum of the MS macronutrients is equal to 4.0 g L^−1^ in fresh CC21 medium (T0W). It dropped to 0.8–1.0 g L^−1^ after 7 wk of culture (Figs. 2*d*, 3*d*). The ammonium, potassium, and calcium ions were consumed at 40 to 60% of their initial concentrations at TW7. The nitrate and sulfate were metabolized at a higher rate, but 5 to 10% of their initial concentrations remained at the same date. Finally, among the sampled macronutrients, only magnesium and phosphorus were completely depleted, which occurred as early as T3W for both clones (Figs. 2*f*, 3*f*).

**Figure 4.**
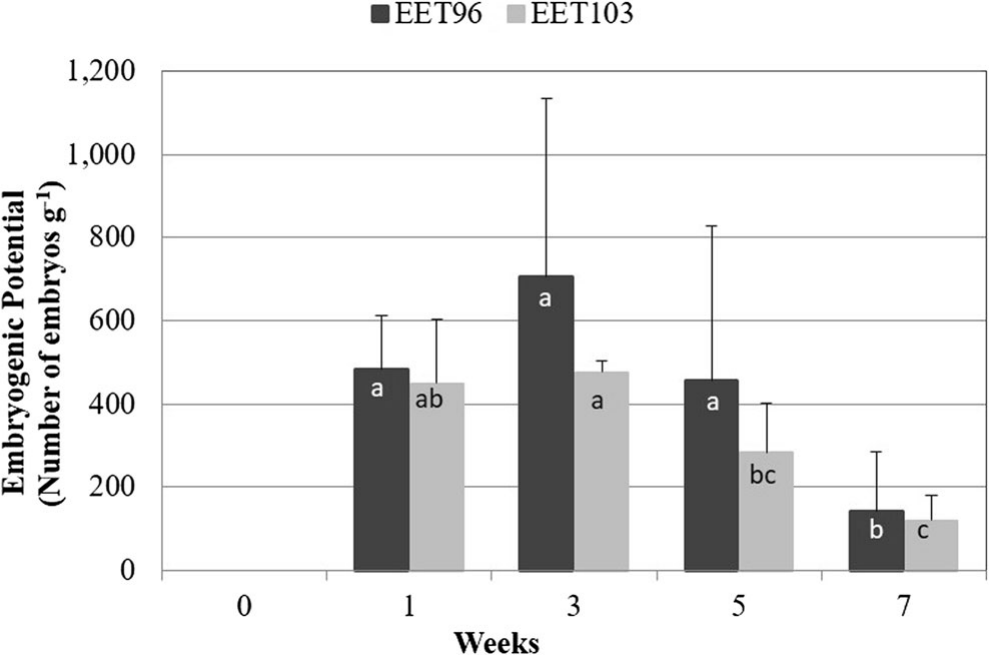
Multiplication step: evolution of the embryogenic potential of *Theobroma cacao* L*.* embryogenic cell lines EET96 and EET103. After various durations of culture in CC21 medium, 0.1 g of callus tissue was transferred into 100 mL CC2 medium. The embryos were counted by the naked eye after 4 wk. The data represent the averages of 16 replicates (four flasks × four measures flask^−1^). Data followed by *different letters* are significantly different at *P* < 5% (Tukey’s test completed separately for each clone).

Evolution of the embryogenic potential of the calluses. The evolution of the tissue qualities within a multiplication cycle in CC21 medium was monitored by measuring the embryogenic potential of the calluses (number of embryos that could be produce g FW^−1^) once transferred to expression conditions without auxin (Fig. 4). A strongly significant effect (*P* < 0.001) of the clones (F = 12.8), and mostly of the duration (F = 39.5) on the embryogenic potential was detected. It reached its highest value after 3 wk of culture, and then significantly decreased at 5 wk for cultivar EET103.

The cocoa embryogenic cell lines were characterized by biphasic growth, a linear growth up to T5W, followed by faster growth up to T7W. The second phase was concomitant with a decrease in the embryogenic capacity of the calluses, which may be due to phosphorus and magnesium deficiencies. Other authors have described such deficiencies using the MS macronutrients during plant cell cultures in liquid medium (Archambault *et al.* 1994; Azevedo *et al.* 2008; Singh and Chaturvedi 2012). It should be noted that the growth cycle was monitored in terms of fresh biomass. The tissues could have a higher WC at the end of the culture period due to the low osmolality of the medium (50 mOsm kgH_2_O^−1^). It is possible that the decrease in the embryogenic potential of the calluses at this time could also be attributed to a higher WC.

Considering these results, a frequency of 3 wk to subculture, the cell lines was selected for the standard procedure. Under this 3-wk regime, the multiplication rate of the biomass ranged from 2 to 3 at each subculture. However, as reported early in the history of plant SE (Smith and Street 1974), a decline of the capacity of the cocoa calluses to regenerate embryos was observed with the number of subculture cycles (data not shown). Therefore, this number was restricted to 10 cycles, a total of 30 wk of culture, in a liquid multiplication medium. In *T. cacao*, the long-term decrease in the embryogenic potential of secondary SE has been associated with an increase of global DNA methylation levels (Rodrίguez Lόpez *et al.* 2010; Adu-gyamfi *et al.* 2016; Quinga *et al.* 2017).

### Expression of the callus embryogenic capacity in liquid medium

**Figure 5.**
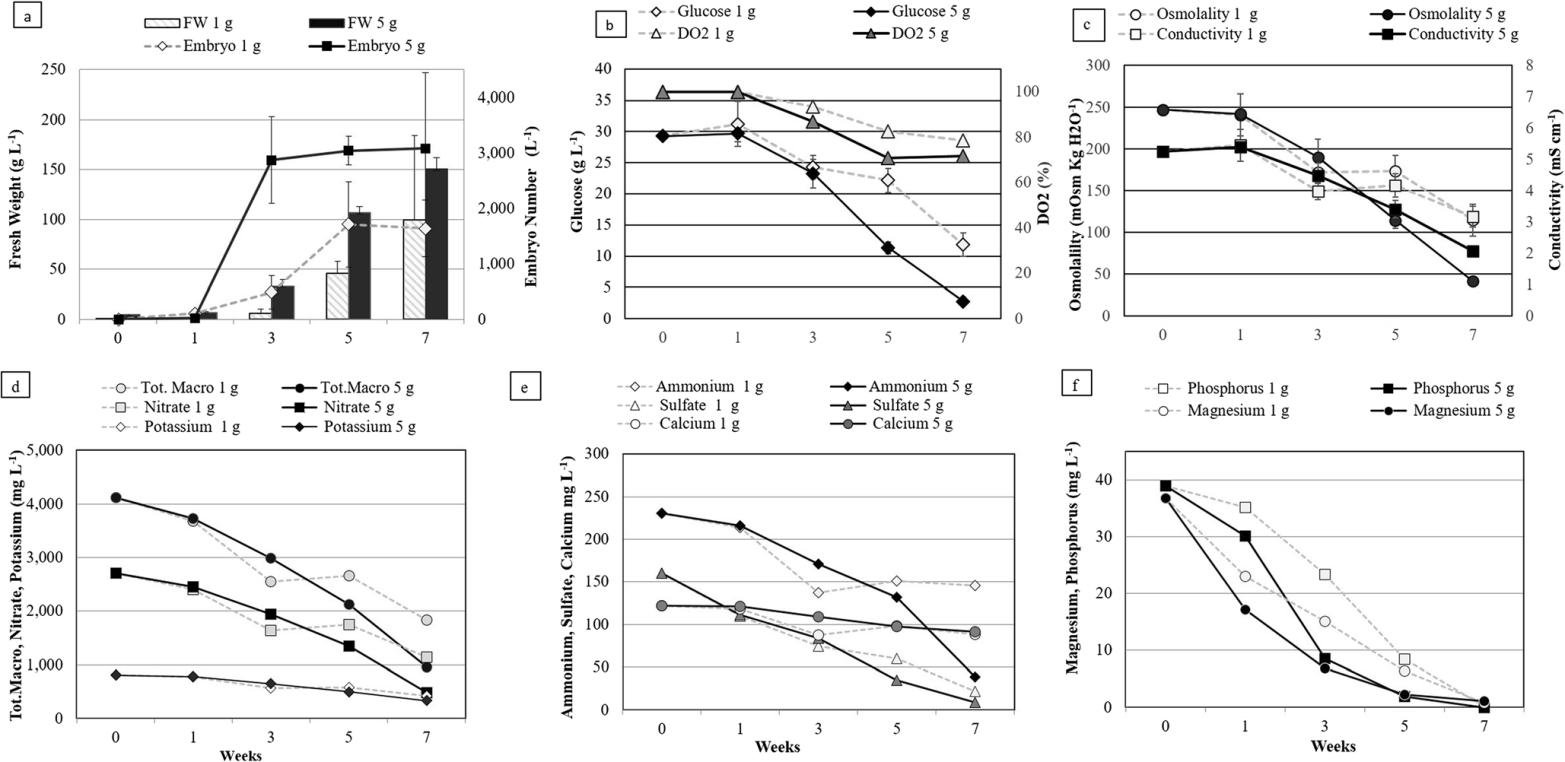
Expression step: growth kinetics of *Theobroma cacao* L*.* cv. EET96 embryo regeneration. The data are the averages of four replicates, each one being a 250-mL flask containing 100 mL of CC2 medium inoculated with an inoculation density of 1 or 5 g L^−1^. Bars represent standard errors unless too small to visualize. For *d*, *e*, and *f*, measurements were taken after combining the spent medium of four flasks.

**Figure 6.**
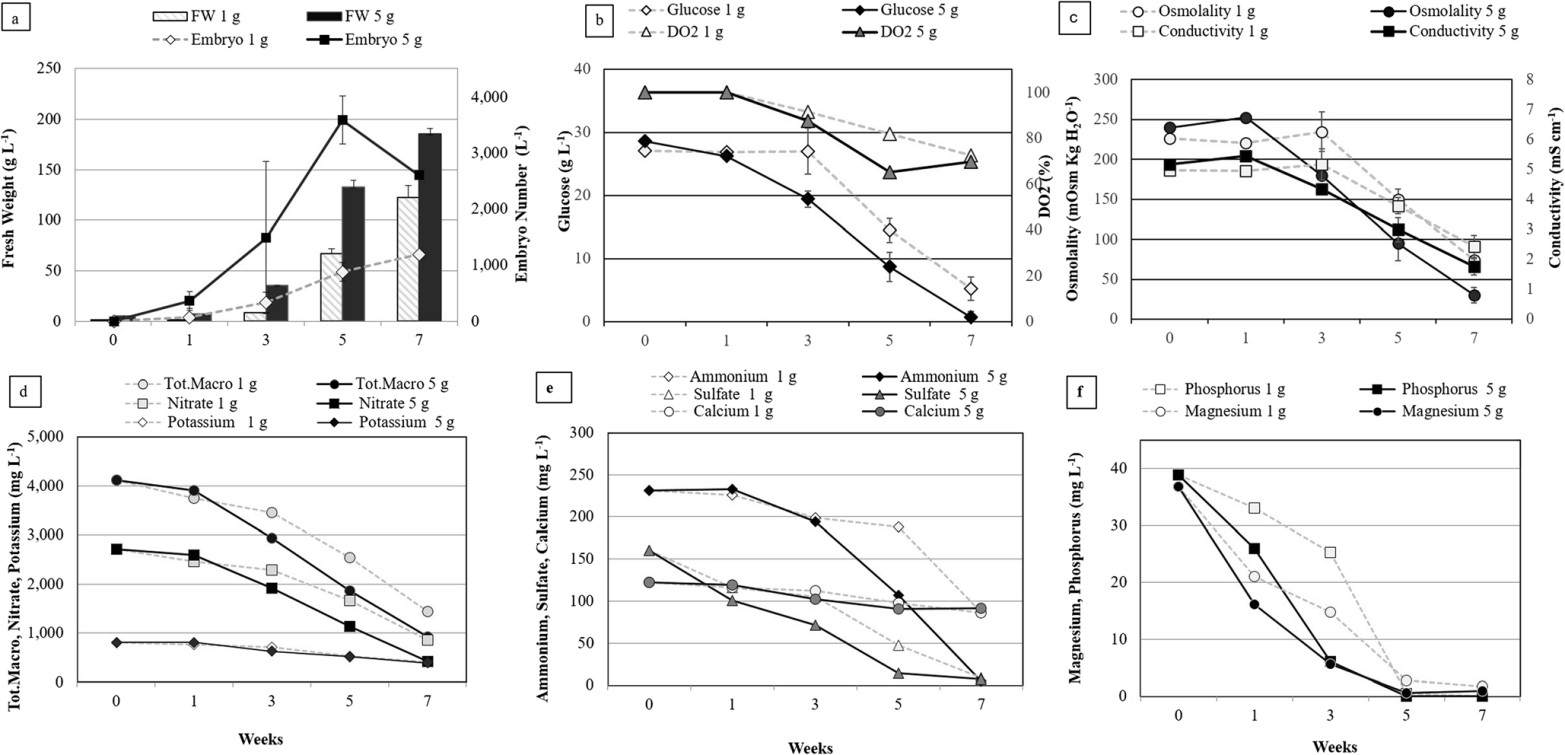
Expression step: growth kinetics of *Theobroma cacao* L*.* cv. EET103 embryo regeneration. The data are the averages of four replicates, each one being a 250-mL flask containing 100 mL of CC2 medium inoculated with an inoculation density of 1 or 5 g L^−1^. *Bars* represent standard errors unless too small to visualize. For *d*, *e*, and *f*, measurements were taken after combining the spent medium of four flasks.

Growth kinetics. Using calluses produced from the cell lines described above, the growth kinetics was compared during the expression step starting with two inoculation densities, 1 and 5 g FW L^−1^ for the clones EET96 and EET103 (Figs. 5 and 6, respectively). The highest concentrations in embryos were reached after 5 wk for the cultures initiated at 5 g FW L^−1^ and ranged between 2500 and 3500 embryos L^−1^ (Figs. 5*a*, 6*a*).

**Figure 7.**
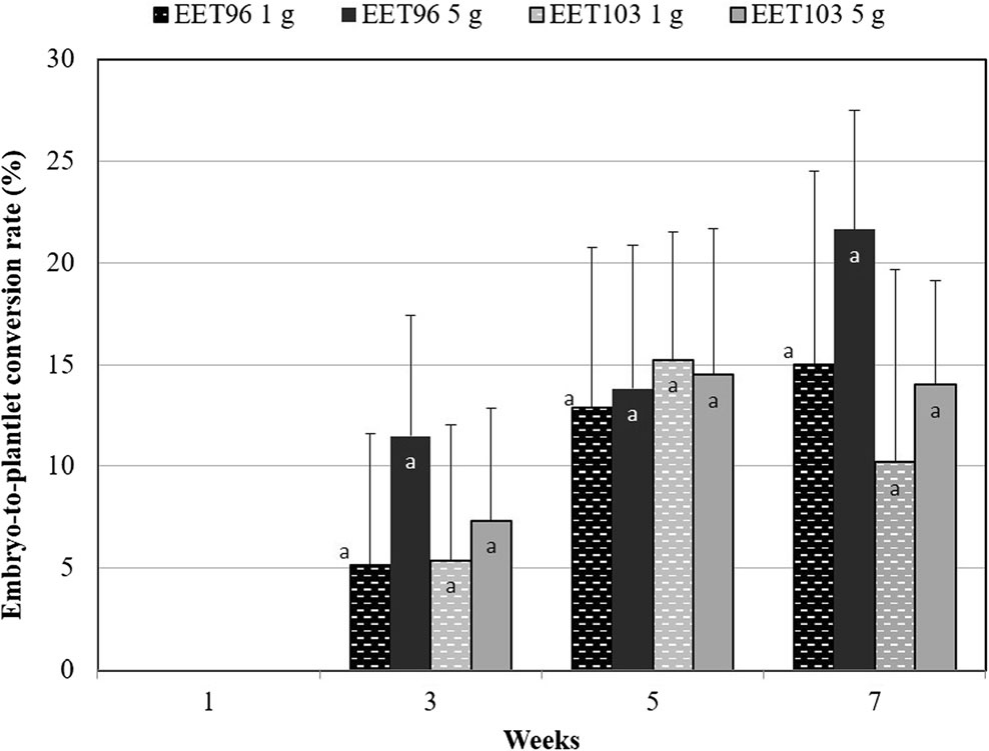
Expression step: evolution of the quality of the *Theobroma cacao* L*.* cvs. EET96 and EET103 embryos. For the embryo-to-plantlet conversion rates, at least 20 embryos per replicate were transferred onto G80 medium and after 3 wk on ENR8 medium. The data (averages of four replicates) followed by *different letters* are significantly different at *P* < 5% (Tukey test completed for each clone separately).

If the embryo concentrations were proportional to the size of the inoculum up to T3W, this was no longer the case after this date. In fact, the highest values of embryogenic potential, expressed as the number of embryos per inoculated gram, were obtained in the cultures inoculated at 1 g FW L^−1^ at T5W for clone EET96 and T7W for clone EET103, and ranged between 1000 and 1500 embryo g^−1^. The embryogenic potential highly depends on the duration (*P* < 0.001, F = 40.9), on the inoculation density (*P* < 0.001, F = 18.2), and to a lesser extent on the genetic background (*P* = 0.021, F = 5.8). The ability of the embryo to develop plantlets, once transferred to maturation followed by a germination medium, tended to increase between T3W and T7W but not significantly (Fig. 7). On the contrary to the embryogenic potential, there was no influence of the inoculation density on the SE quality (*P* = 0.36).

It is not known whether the inhibition of the embryo production in the cultures initiated at 5 g FW L^−1^ resulted from deficiencies in nutrients due to higher consumption rate of nutrients or to decreased tissue osmolality and medium conductivity in the cultures initiated at 5 g FW L^−1^ (Figs. 5*c*, 6*c*). Whatever clone was tested, or the inoculation densities used, the DO_2_ and the NO_3_^−^, K^+^, NH_4_^+^, and Ca^+2^ ions were clearly not limiting factors throughout the kinetics (Figs. 5*d*, *e*, 6*d*, *e*). For clone EET96, inhibition was observed at T5W. At this date, the magnesium and phosphorus were the nutrients with the lowest concentrations, but their levels remained higher than 1.8 mg L^−1^ in both cultures. For clone EET103, inhibition was quantifiable at T7W, and the phosphorus was totally exhausted at T5W in the cultures initiated at 5 g L^−1^. For these cultures, very low concentrations in glucose (0.7 g L^−1^), in Mg^+2^ (0.9 mg L^−1^), and in SO_4_^−2^ (7 mg L^−1^) at T7W were noted.

**Figure 8.**
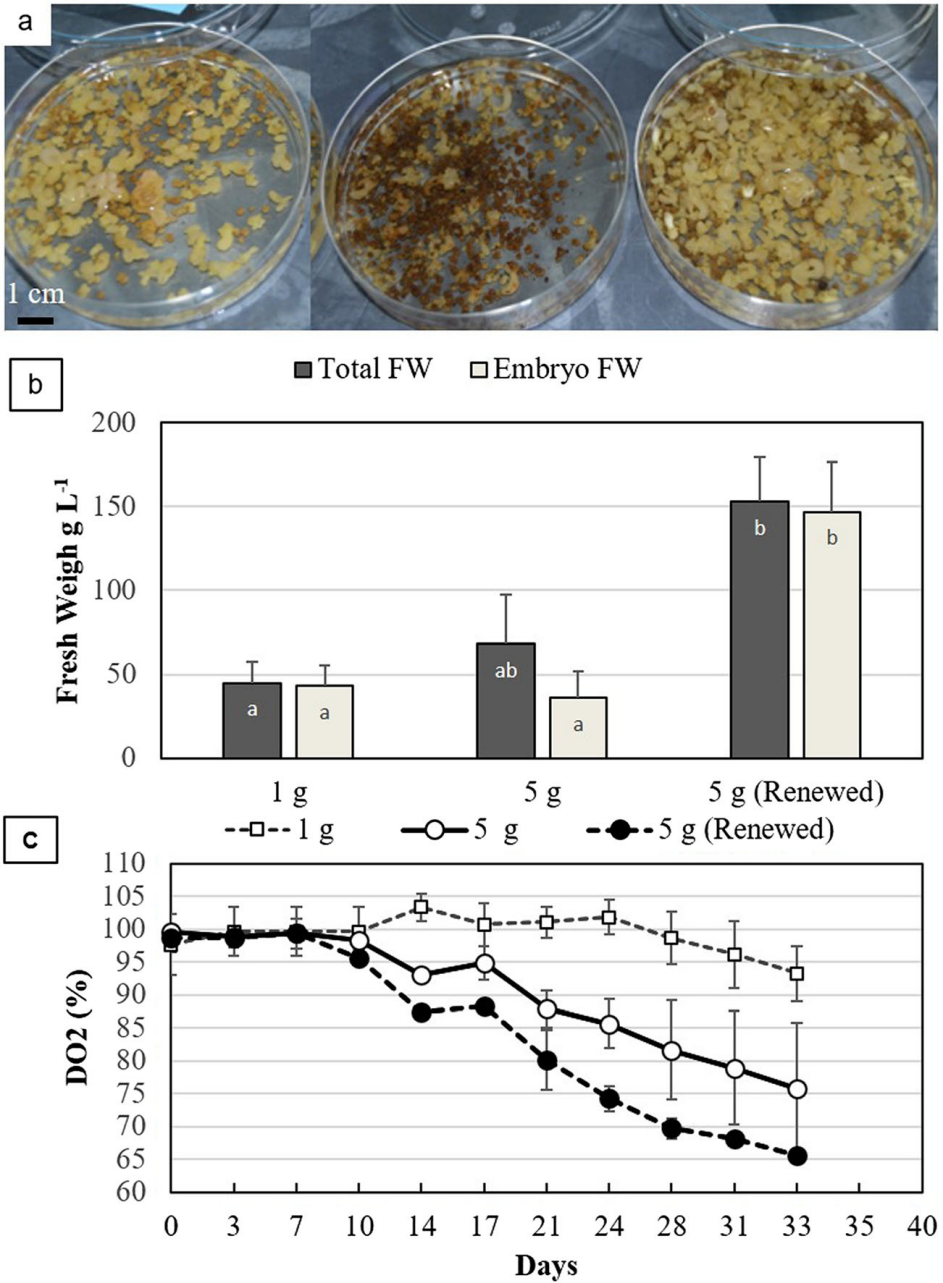
Expression step: effect of the inoculation density and of a twice-weekly medium renewal of *Theobroma cacao* L*.* cv. EET95. (*a*) Embryo suspensions after 5 wk. From left to right: inoculation density of 1 and 5 g L^−1^ with medium renewal. (*b*) Total (whole biomass) and embryo FW at 5 wk. The data (averages of three replicates) followed by different letters are significantly different at *P* < 5% (Tukey test). (*c*) Evolution of the DO_2_. DO_2_ was measured just before the medium was renewed. The data are the averages of three replicates.

Effect of medium renewals. For EET95 cell line, the expression step initiation was compared with 1 and 5 g FW L^−1^, with and without renewing the medium twice a week. Once again, a strong inhibition of the expression was linked to the higher inoculation density (Fig. 8*a*). After 33 d, less than 50% of the biomass was composed of embryos in cultures initiated with 5 g L^−1^, instead of complete differentiation in those initiated at 1 g L^−1^ (Fig. 8*b*). As a consequence of the medium renewals, the inhibition was totally suppressed as the biomass increased to 150 g L^−1^, with 95% of the biomass consisting of embryos. The inhibition of the differentiation at 5 g FW L^−1^ did not result from a limitation in oxygen because the DO_2_ level stayed above 70% (Fig. 8*c*). When the medium was renewed, the DO_2_ dropped to a lower level than without renewal, as early as the 14th day (Fig. 8*c*) (*P* = 0.011, *t* test), which indicated a higher demand for oxygen in the suspensions. This fact suggests that the growth and the differentiation process were hampered before the 21st day, when the expression was started at 5 g FW L^−1^. If this observation does not permit exclusion of nutrient deficiencies as the cause of inhibition, it does provide an argument in favor of the presence of inhibitory compounds released by the tissues.

Based on these results, the standard procedure selected consisted of starting the expression with 1 to 2 g FW L^−1^ inoculum and renewing the medium after 3 to 4 wk of culture. The embryos were collected after three to four additional weeks of growth in liquid medium before direct transfer onto the maturation medium, or after a 3 to 5 wk of development in a temporary immersion bioreactor (Guillou *et al.* 2014).

It was observed that inhibition of the cocoa SE expression was linked to high initial densities, similar to results reported in other plant species like coffee (Zamarripa *et al.* 1991), carrot (*Daucus carota* L.; Kobayashi *et al.* 2000), or larch (*Larix leptolepis* Gordon; Umehara *et al.* 2005). Among the elements monitored, deficiencies in phosphorus and Mg^+2^ are most likely to occur during the expression step, similarly to the multiplication step. However, these ions were consumed more slowly than during the multiplication phase, because they were only completely consumed at T5W or T7W, instead of at T3W. Interestingly, Minyaka *et al.* (2008) reported that supplementing a medium with MgSO_4_ improved the induction and the development of secondary SE on solid medium. Optimal cocoa SE probably requires these elements in higher concentrations than the elements provided by the MS basal medium commonly used in plant cell cultures. Nevertheless, results are insufficient to determine a definitive role of nutrient depletion on the inhibition of cocoa SE production. Generally, to the naked eye, embryos are smaller in the cultures initiated at 5 g FW L^−1^ as early as the third week at which time the concentrations of phosphorus and the magnesium were clearly not yet limited. This point was confirmed by the experiment that renewed the medium twice weekly. The demand in oxygen in the high-density cultures was hampered before the end of the 3rd week. Taken together, these observations suggest that this inhibition was most likely associated with the diffusion of SE self-inhibitors from the tissues into the medium, similar to those identified in other species, such as 4-hydroxybenzyl alcohol in carrot (Kobayashi *et al.* 2000), or vanillyl benzyl ether in larch (Umehara *et al.* 2005).

Effect of an increase in the tissue osmolality. Myo-inositol is known to be involved in seed development, seed desiccation, signaling pathways, cell wall biosynthesis, and various stress-related responses (Loewus and Murthy 2000). For this reason, myo-inositol was tested as an osmotic agent rather than other sugar alcohols, such as mannitol or sorbitol, in order to produce higher frequencies of cocoa SE conversion into plantlets.

**Figure 9.**
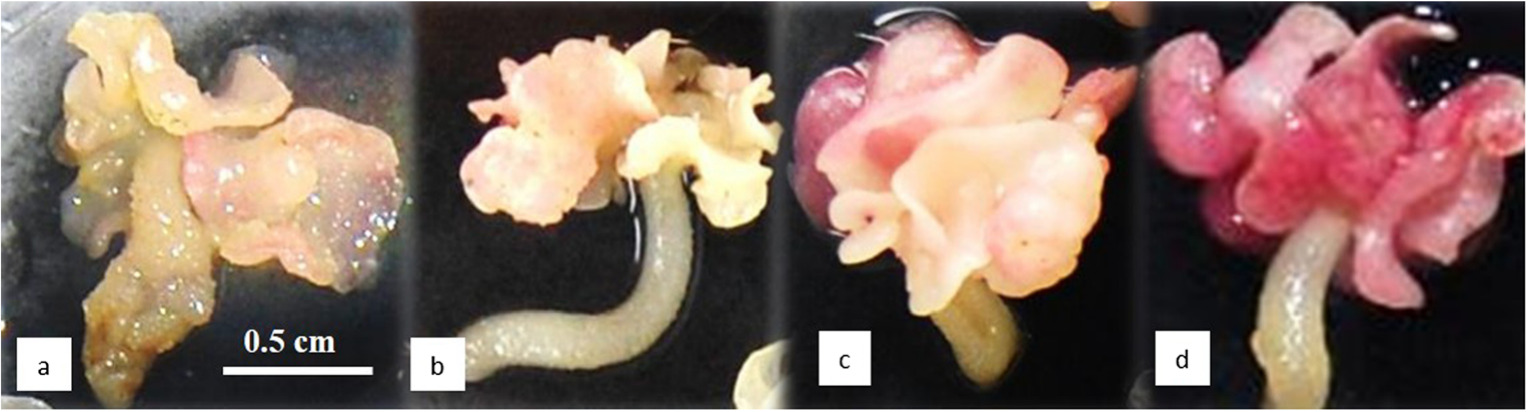
Expression step: effect of supplementing CC2 medium with different myo-inositol concentrations on *Theobroma cacao* L*.* cv. EET103 embryos after 4 wk. (*a*) Embryos in control CC20 medium without myo-inositol; (*b*) embryos in CC2 medium with 15 g L^−1^ of myo-inositol; (*c*) embryos in CC2 medium with 30 g L^−1^ of myo-inositol; (*d*) embryos in CC2 medium with 45 g L^−1^ of myo-inositol.

In the first experiment, the growth and the embryo formation were partially inhibited with myo-inositol at 30 and 45 g L^−1^ (Table 2). After 30 d, the embryo WC decreased from 90% in the control to 82%, with the highest myo-inositol concentration. When the tissue osmolality was increased, the morphology of the embryos changed. They showed a higher degree of opacity, and more elongated hypocotyls with anthocyanin pigmentation (Fig. 9). After culture on a maturation medium followed by a germination medium, the aptitude of the embryos to develop normal plantlets was improved proportionally to the myo-inositol concentration, from 16 to 33% with myo-inositol at a concentration of 45 g L^−1^ (Table 2).

**Table 2.** Effect of myo-inositol supplementation during the expression step on the embryo quality of *Theobroma cacao* L*.* cv. EET103. The expression step was initiated with an inoculation density of 5 g L^−1^ in 250-mL flasks containing 100 mL of CC2 medium. Fresh weight (FW), embryo number, and water content (WC) were measured after 30 d of expression (data are the averages of five replicates). The embryo-to-plantlet conversion rates were measured after 3 wk on maturation medium and 8 wk on germination medium (data are the averages of five replicates of at least 20 embryos). (± are the standard errors, data followed by *different letters* are significantly different according to the Tukey test (*P* < 5%))

Glucose	Myo-inositol	Molarity	Osmolality fresh medium	FW	Embryo number	WC	Embryo-to-plantlet conversion rate
g L^−1^	g L^−1^	M	mOsm kg H_2_O^−1^	g L^−1^	Number L^−1^	%	% Normal
30	0	0.17	261	65 ± 27 a	1114 ± 131 a	90.2 ± 2.7 a	16.4 ± 2.1 a
30	15	0.25	337	71 ± 13 a	876 ± 191 ab	88.9 ± 0.5 ab	25.4 ± 1.9 ab
30	30	0.33	441	45 ± 21 ab	584 ± 242 bc	82.7 ± 3.5 bc	27.7 ± 2.3 b
30	45	0.42	542	32 ± 7 b	272 ± 118 c	82.5 ± 1.1 c	33.0 ± 3.2 b

Therefore, in the second experiment, the medium was supplemented with myo-inositol at 50 g L^−1^ once the embryos reached the early torpedo-stages (after 4 wk of culture). After an additional 4 wk, the increase in the fresh-medium myo-inositol concentration from 10 to 45 g L^−1^ led to an increase in the embryo osmolality, from 252 to 600 mOsm kgH_2_O^−1^ for SCA6, and from 304 to 502 for EET103 (Table 3). This coincided with slight but statistically significant tissue WC decreases of 6%. This treatment was followed by significantly higher conversion rates into normal plantlets, with a rate of 48.3% instead of 13.3% for SCA6, and 40.8% instead of 15.8% for EET103. Concomitantly, the rates of conversion into abnormal plantlets were reduced. The incidence of this supplementation in medium in which the glucose was replaced by sucrose was also checked. In this case, this sucrose treatment led to a significant increase of the embryo quality for SCA6, but not for EET103. When the sucrose concentration was increased from 30 to 80 g L^−1^, the embryo quality tended to improve though not significantly for both clones, despite the fact that this treatment reduced their WC.

**Table 3.** Effect of an increase of the osmolality during the last week of the expression step on the quality of the embryos cvs. SCA6 and EET103. After 4 wk of expression on CC2 medium containing glucose at 30 g L^−1^, embryos were cultured (50 embryos 100 mL^−1^) four additional wk in CC2 supplemented or not with myo-inositol or sucrose at different concentrations. The embryos were subcultured on maturation medium during 3 wk then 8 wk on germination medium. The embryo water content (WC) and osmolality were measured at the end of the expression (data are the averages of four replicates of 30 embryos for WC, of four replicates of 10 embryos for the osmolality). For the conversion rate, data are the averages of four replicates of 30 embryos. (± are the standard errors, data followed by *different letters* are significantly different according to the Tukey test (*P* < 5%))

Clone	Glucose	Sucrose	Myo-inositol	Molarity	Osmolality fresh medium	Osmolality embryos	WC	Embryo-to-plantlet conversion rate	
	g L^−1^	g L^−1^	g L^−1^	M	mOsm kg H_2_O^−1^	mOsm kg H_2_O^−1^	%	% Normal	% Abnormal
SCA6	30			0.17	274	252 ± 15 d	92.4 ± 0.4 a	13.3 ± 7.7 cd	67.5 ± 12.9 a
	30		50	0.44	556	600 ± 29 a	86.3 ± 0.5 c	48.3 ± 7.9 ab	45.8 ± 9.2 ab
		30		0.09	193	314 ± 15 c	91.7 ± 0.2 b	9.2 ± 9.9 b	63.3 ± 7.2 a
		30	50	0.37	468	600 ± 17 a	86.0 ± 0.6 d	61.7 ± 13.5 a	32.5 ± 10.3 b
		80		0.23	373	557 ± 7 b	87.4 ± 0.2 c	33.3 ± 8.2 bc	55.5 ± 10.7 a
EET103	30			0.17	274	304 ± 5 c	92.6 ± 0.1 a	15.8 ± 5.7 b	71.6 ± 5.8 a
	30		50	0.44	556	502 ± 15 ab	86.7 ± 0.4 c	40.8 ± 6.3 a	50.8 ± 5.7 b
		30		0.09	193	252 ± 65 d	92.1 ± 0.3 b	15.8 ± 3.2 b	57.5 ± 11.1 ab
		30	50	0.37	468	533 ± 26 a	85.5 ± 0.6 d	26.7 ± 6.1 ab	61.6 ± 11.7 ab
		80		0.23	373	472 ± 13 b	86.7 ± 0.8 c	25.0 ± 16.4 ab	62.5 ± 7.9 ab

As observed by Niemenak *et al.* (2015), a treatment with a high sucrose concentration modifies the appearance of the embryos, which indicates a higher degree of maturation. However, myo-inositol was more efficient than sucrose for increasing the SE quality. Increasing the tissue osmolality generally inhibited the formation of new embryos from micro-calluses attached to the embryos. Consequently, the embryo populations appeared more uniform at the end of the expression step. The comparison of the effect of myo-inositol with other osmotic agents (mannitol, sorbitol, and maltose), with identical molarity, and the continuation of these treatments beyond the expression stage such as during maturation on solid medium, is currently underway.

**Figure 10.**
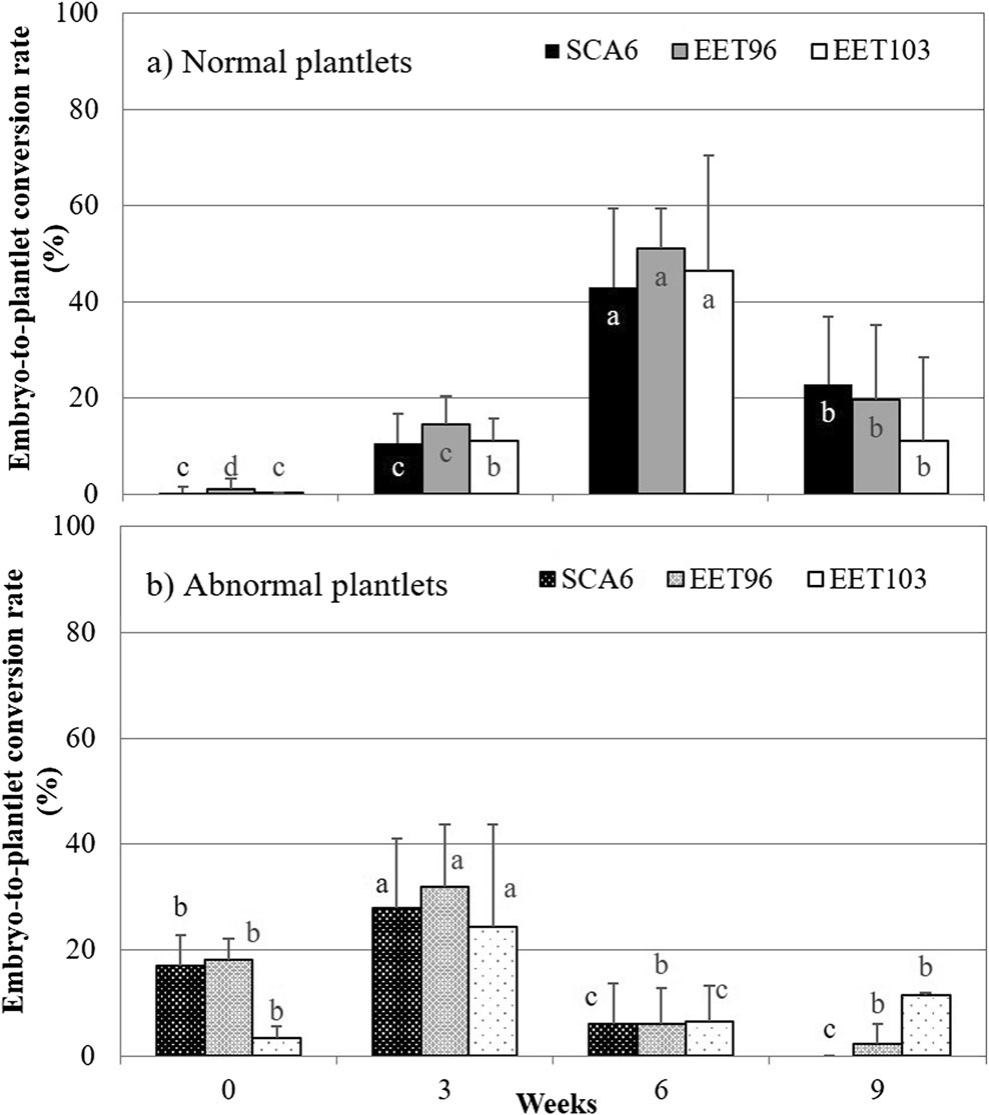
Effect of the duration of culture on G80 maturation medium on the conversion into normal or abnormal plantlets *Theobroma cacao* L*.* cvs. SCA6, EET96, and EET103. Selected embryos were subcultured on G80 maturation medium in 90 × 20-mm Petri dishes (40 embryos per dishes). For their conversion into plantlets, they were transplanted onto ENR8 germination medium. The conversion rates were measured after 8 wk. Data (averages of 10 replicates of 40 embryos) followed by *different letters* are significantly different according to Tukey test completed separately for each clone (*P* < 5%).

**Figure 11.**
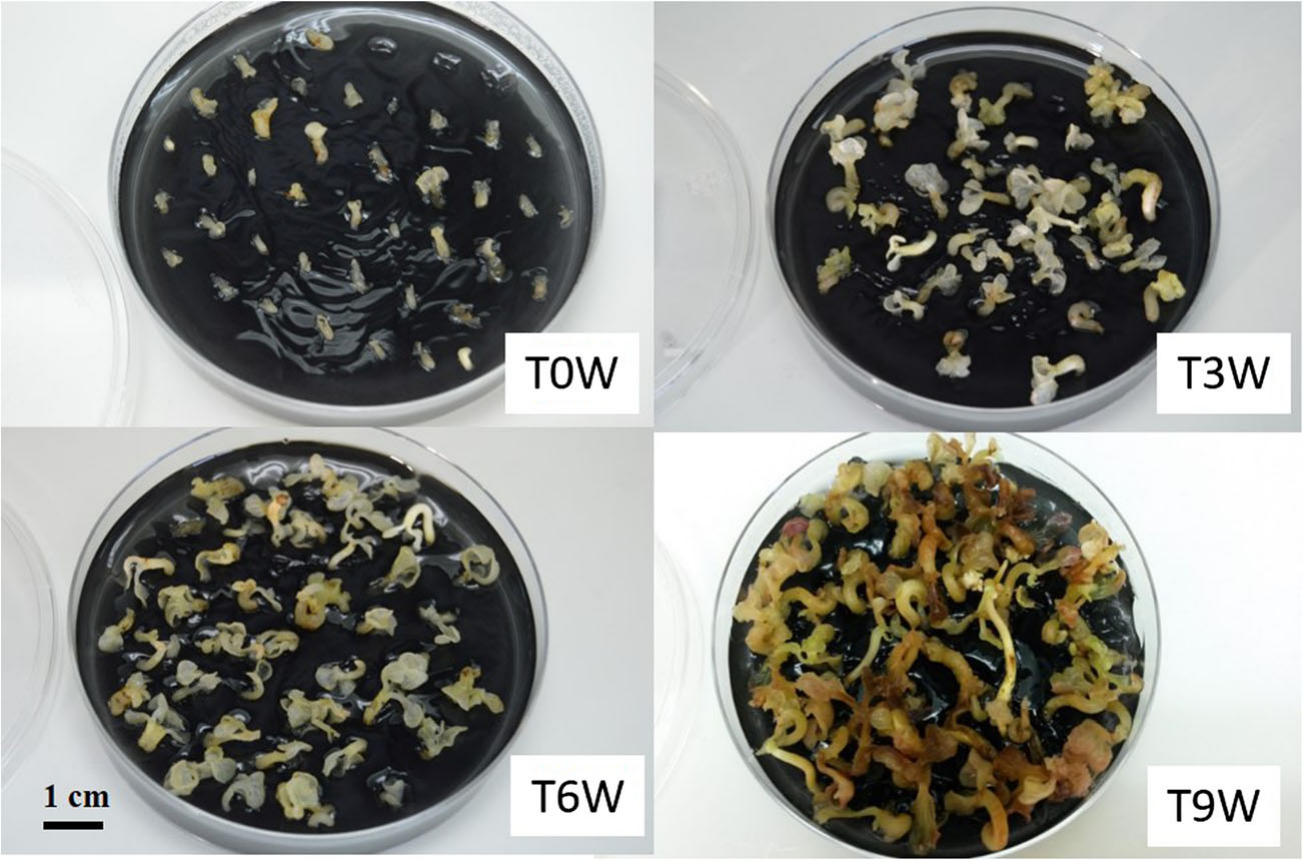
Evolution of the embryos during the maturation step of *Theobroma cacao* L*.* cv. EET96. T0W, embryos after the harvest without maturation phase; T3W, embryos after 3 wk on maturation medium; T6W, embryos after 6 wk on maturation medium; T9W, embryos after 9 wk on maturation medium.

Embryo maturation on solid medium. The embryo-to-plantlet conversion rates were compared for clones SCA6, EET96, and EET103 after 0, 3, 6, and 9 wk of maturation on G80 medium (Table 1). When the embryos were directly transferred from the CC2 expression medium onto the ENR8 germination medium (T0W), their conversion into normal plantlets was practically nil (Fig. 10). For the three clones SCA6, EET96, and EET103, the best conversion rates (43, 51, and 47%, respectively) were observed after 6 wk of maturation. On average for the three clones, the frequency of regeneration into normal plantlets increased from 13 to 47% when the maturation duration was extended from 3 to 6 wk. Meanwhile, the regeneration into abnormal plantlets dropped from 31 to 6%. After 9 wk of maturation, the embryo quality decreased. During this kinetic period on maturation medium, neither the osmotic potential of the embryos nor their WC changed (data not shown). The WC never decreased below 90%, particularly between T3W and T6W (Fig. 11 shows the evolution of the appearance of the embryos). The higher quality after 6 wk of maturation could not be correlated with a greater opacity, or any other signs of maturation such as a partially dehydrated appearance. However, the embryos started to present signs of oxidation after 9 wk, which could be responsible for a lower quality at this period.

The positive effect of the maturation step could come from certain components of the G80 medium, and most probably from active charcoal and/or from ABA, which are both present at 1 g L^−1^ and 1 mg L^−1^, respectively. Active charcoal is known to trap toxic compounds such as residual or endogenous auxins, which affects the SE quality in soybean (Buchheim *et al.* 1989). The combination of charcoal and ABA at 5 to 25 mg L^−1^ improved the production of well-shaped SE in coniferous species, despite the fact that ABA was trapped by charcoal (Pullman *et al.* 2005). Free ABA was indeed undetectable in the G80 medium in the present experiment (data not shown)*.* Finally, the hypothesis that the positive impact of the maturation on the SE quality does not result from the G80 medium itself, but from the microenvironment provided by Petri dishes cannot be excluded. This hypothesis is currently being investigated. In spruce, a confined environment induced a larger number of well-formed mature embryos and a lower rate of precocious germination than a ventilated environment, and the addition of 5% CO_2_ or 10% O_2_ in a vented environment promoted SE (El Meskaoui and Tremblay 1999; El Meskaoui *et al.* 2006).

## Conclusions

This is the first detailed report describing the establishment of cocoa embryogenic cell lines and their growth kinetics in liquid medium. This protocol is based on obtaining and rigorously selecting HFSE callus. The main points necessary to succeed in establishing the cell lines are as follows: (1) at the beginning of the process, the primary embryos used as explants must be taken at the torpedo-stage to obtain HFSE callus, and (2) particular attention must be focused on the inoculation densities of the suspensions, as in the case of Robusta coffee (Ducos *et al.* 2007), because high densities hamper the precocious embryo differentiation, which tends to occur despite the presence of the 2,4,5-T in the medium.

Once placed in the optimal expression conditions in flasks, 1 g of callus produced 1000 to 1500 embryos. Compared to certain other species, the resulting embryogenic potential is relatively low. Productivity levels of over 5 × 10^5^ embryos g^−1^ of callus have been frequently reported in carrot, up to 2 × 10^5^ embryos g^−1^ of callus in Robusta coffee (Zamarripa *et al.* 1991), and 0.4 to 0.8 × 10^5^ in Arabica coffee (Van Boxtel and Berthouly 1996; De Feria *et al.* 2003, Etienne 2005). The frequent occurrence of abnormal cocoa plantlets with shoots continuously producing cotyledon-like leaves and long internodes was noted, which is a phenomenon that has been reported by other authors (Maximova *et al.* 2005; Quainoo and Dwomon 2012). Two paths were identified for improving the quality of cocoa SE regenerated in liquid medium. One was to increase the osmolality during the expression step and the second was to extend the maturation step in Petri dishes. Interestingly, both treatments increased the rate of conversion into normal plantlets while they decreased the conversion into abnormal plantlets, which indicated that the morphological issue was not the consequence of somaclonal variation, but most likely due to physiological disorders resulting from incomplete maturation.

One of the important strategies for future improvements of the quality of the embryogenic cocoa tissues, including the embryogenic potential of the callus at the end of multiplication, and the capacity of the embryos to regenerate plants at the end of the expression, consists of implementing these steps in a bioreactor system that continuously monitors and controls the environmental conditions. There are some studies that indicated SE optimization in a stirred bioreactor by controlling the DO_2_ (Archambault *et al.* 1994; De Feria *et al.* 2003; Shimazu and Kurata 2003).

In the framework of the Nestlé Cocoa Plan, the method described in this study was used to regenerate 260,000 plants, which, after their acclimation, have been sent to producing countries, mainly Ecuador and Ivory Coast (Guillou *et al.* 2014). The Indonesian Coffee and Cocoa Research Institute (ICCRI) scaled up its production to deliver 74 M cocoa somatic seedlings during the period from 2009 to 2011 and modified certain steps for particular genotypes (Sena Gomes *et al.* 2015). To address the issues of cocoa production by diffusing more productive varieties with resistance to biotic and abiotic stresses, the application of HFSE will be of interest for future breeding programs, and for mass propagation of validated planting materials.
